# Adherence to a Healthy Nordic Food Index Is Associated with a Lower Risk of Type-2 Diabetes—The Danish Diet, Cancer and Health Cohort Study

**DOI:** 10.3390/nu7105418

**Published:** 2015-10-21

**Authors:** Sandra Amalie Lacoppidan, Cecilie Kyrø, Steffen Loft, Anne Helnæs, Jane Christensen, Camilla Plambeck Hansen, Christina Catherine Dahm, Kim Overvad, Anne Tjønneland, Anja Olsen

**Affiliations:** 1 Danish Cancer Society Research Center, Strandboulevarden 49, 2100 Copenhagen, Denmark; sanlac@cancer.dk (S.A.L.) ceciliek@cancer.dk (C.K); akhj@cancer.dk (A.H.); jane@cancer.dk (J.C.); annet@cancer.dk (A.T.); anja@cancer.dk (A.O.); 2 Department of Public Health, Section of Environmental Health, Faculty of Health and Medical Sciences, University of Copenhagen, Øster Farimagsgade 5, 1014 København K, Denmark; stl@sund.ku.dk; 3 Department of Public Health, Section for Epidemiology, Aarhus University, Bartholins Allé 2, 8000 Aarhus C, Denmark; cph@ph.au.dk (C.P.H.); ccd@ph.au.dk (C.C.D.); ko@ph.au.dk (K.O.)

**Keywords:** Type-2 diabetes (T2D), Nordic diet, dietary pattern, prospective cohort.

## Abstract

Background: Type-2 diabetes (T2D) prevalence is rapidly increasing worldwide. Lifestyle factors, in particular obesity, diet, and physical activity play a significant role in the etiology of the disease. Of dietary patterns, particularly the Mediterranean diet has been studied, and generally a protective association has been identified. However, other regional diets are less explored. Objective: The aim of the present study was to investigate the association between adherence to a healthy Nordic food index and the risk of T2D. The index consists of six food items: fish, cabbage, rye bread, oatmeal, apples and pears, and root vegetables. Methods: Data was obtained from a prospective cohort study of 57,053 Danish men and women aged 50–64 years, at baseline, of whom 7366 developed T2D (median follow-up: 15.3 years). The Cox proportional hazards model was used to assess the association between the healthy Nordic food index and risk of T2D, adjusted for potential confounders. Results: Greater adherence to the healthy Nordic food index was significantly associated with lower risk of T2D after adjusting for potential confounders. An index score of 5−6 points (high adherence) was associated with a statistically significantly 25% lower T2D risk in women (HR: 0.75, 95%CI: 0.61–0.92) and 38% in men (HR: 0.62; 95%CI: 0.53–0.71) compared to those with an index score of 0 points (poor adherence). Conclusion: Adherence to a healthy Nordic food index was found to be inversely associated with risk of T2D, suggesting that regional diets other than the Mediterranean may also be recommended for prevention of T2D.

## 1. Introduction

Type-2 diabetes (T2D) is a chronic metabolic disease with an increasing global incidence and prevalence. Around 4 percent of the population worldwide were estimated to have diabetes in 2000, but the number is projected to increase to 4.4% or 366 million people by 2030 [1]. The majority of the cases are onset at age 40–59 and are expected be type 2 diabetes [1,2]. It is well established that lifestyle plays a significant role in the etiology of T2D [3,4]. Among lifestyle factors, overweight and obesity particularly increase the risk of T2D. Diet also plays a role in relation to T2D risk [3]. Several studies have investigated the effect of different dietary exposures in relation to development of T2D, and intakes of whole grains, dietary fibre, root vegetables, and green leafy vegetables have been suggested to be inversely associated with risk of T2D [5,6,7]. 

In nutrition research, focus has previously mainly been on single foods or nutrients. However, people consume meals consisting of combinations of numerous nutrients and foods. Hence, it is relevant to examine a combination of a variety of foods in relation to T2D. Different dietary indices, such as the Mediterranean diet, have been investigated in relation T2D, in Mediterranean, American as well as other European populations [8,9,10,11,12]. Compliance with this dietary pattern is low in non-Mediterranean countries, maybe because non-Mediterranean populations may find it difficult to comply with a diet which is foreign to them [6,13]. It might therefore be relevant to investigate health effects of other regional diets such as the Nordic diet within a Nordic population. It is possible that other regional diets also carry health benefits, and it might be easier for a population to comply with a diet that is familiar to them instead of adapting to a “foreign diet” [14]. Intervention studies have suggested that a healthy Nordic diet improves markers of T2D risk, such as body weight and insulin sensitivity [15,16,17]. A small prospective study found no association between adherence to a health Nordic diet and risk of T2D [18], but due to the small study size (total cohort *n* < 7000, cases = 541) results from larger prospective studies are needed. This aim of the study was to investigate the association between a healthy regional Nordic diet and risk of T2D in a large prospective cohort study.

## 2. Method and Material

### 2.1. Study Population

From December 1993 to May 1997, a total of 160,725 subjects (80,996 men and 79,729 women) were invited to participate in the Danish cohort study: the Diet, Cancer, and Health (DCH) cohort [19]. The criteria for invitation were: aged 50–64 years, born in Denmark, no diagnosis of cancer (registered in the Danish Cancer Registry), and living in greater Copenhagen and Aarhus areas at baseline. Unique personal identification numbers assigned to all Danish citizens from the Civil Registration System (CPR) were used to identify potential participants [20]. The DCH cohort had a 35% overall participation, comprising a total of 57,053 eligible subjects [19]. The cohort was approved by the regional ethical committees on human studies in Copenhagen and Aarhus and by the Danish Protection Agency. Moreover, written informed consent was obtained from all participants. 

Of the 57,053 participants, 1766 were excluded, due to diagnosis of cancer and/or T2D before baseline. Furthermore, participants with missing information on the exposure variables of interest in this study (*n =* 55) or potential confounders (*n =* 149) were excluded. Finally, subjects diagnosed with diabetes before 1st January 1995 were excluded (*n =* 15) as well as persons that were included and deceased before this date (*n =* 8), because the National Diabetes Registry is only well defined for subjects entering after this date (see “*case ascertainment and selection*”) [21]. Thus, leaving 55,060 eligible subjects (28,953 women and 26,107 men) for the present study. 

### 2.2. Case Ascertainment and Selection

There were 7366 incident cases of diabetes (3269 women and 4097 men) identified from the National Diabetes Registry (NDR) [21]. The NDR was established in 2006 by the National Board of Health by linking nationwide registries. Because of different dates of initiation of the underlying registers and accumulation of prevalent cases, dates of inclusion into the NDR has been found to be well defined only for persons entering after 1st January 1995, even though inclusion of cases started at 1st January 1990) [21,22]. Subjects are registered in NDR if they are classified as having diabetes with a date of inclusion equal to the earliest of the dates where one of the following criteria were met: Diagnosis of diabetes in the National Patient Registry; Chiropody for diabetic patients; Five blood glucose measurements within one year; Two blood glucose measurements per year in five consecutive years; Second purchase of oral glucose-lowering drugs within six months; Second purchase of prescribed insulin. As the register is based on administrative records, the date of inclusion can only be taken as a proxy for the date of diagnosis. 56.6% of the cases included in the present study met more than one of the criteria. 

NDR does not distinguish between cases of type-1 diabetes (T1D) and T2D. Since the present study is investigating a middle-aged population, and thus it is assumed that the entire incident cases of diabetes registered in NDR can be attributed T2D. Moreover lifestyle behaviour and diet habits are associated with T2D risk and not believed to influence T1D risk, the present study therefore exclusively focuses on T2D. 

All 55,060 cohort participants were followed from baseline until censoring, which was the date of diagnosis of diabetes, date of death, date of emigration, or end of follow-up (31st December 2011), whichever came first. Date of death and date of emigration was identified from the CPR. Baseline was defined as the data of visiting the center, however, with the exception of participants who visited the center before 1st January 1995. For those, baseline was set to 1st January 1995. 

### 2.3. Dietary Assessment

Prior to attendance in the study, the participants completed a 192-item FFQ, which has been validated previously [23,24]. In the questionnaire, the participants were asked to report their average intake within the last 12 months in twelve categories ranging from never to more than eight times a day. The intake of specific foods and nutrients was calculated for each participant by the software program FoodCalc [25], using specifically developed standardized recipes and portions sizes. The FFQs were processed by optical scanning and checked for missing values and reading errors. The errors were clarified and corrected by trained professionals before the participants left the study center [19]. 

Food items of the healthy Nordic diet index included in the study as exposure variables were: fish, cabbage, rye bread, oatmeal, apples and pears, and root vegetables. The index has previously been defined and the foods included in the index were chosen *a priori* based on the following criteria: (1) that they had to have an anticipated health benefit; (2) information on intake should be obtainable from the FFQ; (3) they had to originate from the Nordic nature, and (4) they should still hold a quantitative role the Nordic diet [26]. Information on intake of fish, cabbage, apples and pears, and root vegetables was derived from multiple questions in the FFQ, whereas information on oatmeal and rye bread originated from one question each. Fish intake was based on 23 questions regarding a variety of fish consumed as hot meals or in sandwiches, while cabbages intake was derived from six questions of different cabbage types (cauliflower, Brussels sprouts, broccoli, kale, white cabbage, and red cabbage). Information about apple and pear intake was collected from two questions: one on apple and one on pear intake. Intake of root vegetables was collected from several questions concerning intake of raw and cooked root vegetables separately, as well as part of recipes. In the DCH cohort, carrot was the main root vegetable consumed. 

The healthy Nordic index is developed in accordance with the original Mediterranean Diet score constructed by Trichopoulou and colleagues [27,28]. Thus, for each of the six Nordic food items, one point was given for intake equal to or greater than the sex-specific median. As information on both oatmeal and rye bread intake was obtained from only one question each, it was not possible to use medians as cut-off values. Thus, the cut-off values for oatmeal and rye bread were defined using sex-specific spline curves with boundaries at predefined questionnaire categories. One point was given for an intake of the following: Men: fish ≥ 42 g/day, cabbage ≥ 14 g/day, oatmeal ≥ 21 g/day, rye bread ≥ 113 g/day, apples and pears ≥ 56 g/day, and root vegetables ≥ 16 g/day. Women: fish ≥ 35 g/day, cabbage ≥ 16 g/day, oatmeal ≥ 21 g/day, rye bread ≥ 63 g/day, apples and pears ≥ 71 g/day, and root vegetables ≥ 28 g/day. A score of either 0 or 1 was given for each item, allowing each participant to score between 0 (lowest adherence) and 6 (highest adherence).

### 2.4. Statistical Analysis

The association between the healthy Nordic food index and T2D was estimated using Cox proportional hazards models using age as the underlying time scale, and expressed as hazard ratios (HR) with corresponding 95% confidence intervals (CI). Time under study was included as a linear spline with boundaries at one, two, and three years after entry into the study. All P-values were two-sided, and the statistical significance level was set to 0.05. 

Interaction between the healthy Nordic food index and sex was investigated, and significant interaction was identified (*p* = 0.0186). Consequently, all analyses were stratified by sex. 

The healthy Nordic food index was assessed as a linear variable (1-point unit) and as a categorical variable (0–6 points). The reference category comprised individuals with the poorest adherence to the index (0 points). The highest adherence categories, 5 and 6 points were combined to one category (5–6 points), due to few cases in the category rating “6 points”. 

All estimates are presented as both crude (model 1) and adjusted (models 2, 3, and 4). In model 2, adjustments were made for the following potential confounding lifestyle factors: alcohol (abstainers and drinkers with different boundaries set for men and women), smoking status (never, former, current), schooling level (low: ≤7 years, medium 8–10 years, and high: ≥10 years), participation in sports (yes/no), intake of meat (g/day of red and processed meat). In model 3, adjustment was additionally done for total energy intake (kJ/day) using the standard multivariate method [29]. Model 4 was additionally adjusted for two potential mediators: waist circumference (WC) and body mass index (BMI). In order to preserve statistical power, we adjusted for physical activity using the indicator variable “Participate in sports (yes/no)”. We also had several other measures of physical activity, but adding them to the model did not change the results considerably.

Linearity of continuous variables were evaluated graphically by linear splines with three or nine knots placed at quartiles or deciles among cases. The healthy Nordic food index, BMI, WC, and intake of red and processed meat were found to be linearly associated with T2D, whereas alcohol intake was related to T2D risk with an apparently U-shaped pattern. To ensure proper adjustment accounting for this non-linear association, alcohol intake among drinkers was included as linear splines with boundaries: alcohol intake 12 g/day for women and 24 g/day for men, respectively. 

For the analyses evaluating the individual association between T2D and each of the six food items included in the index, the same confounder adjustments were made, but further adjustments for the remaining five food items (mutually adjustment) were performed. 

A sensitivity analysis was conducted including only “confirmed cases”. It is possible that the register may have included some non-diabetics as cases. Two of the inclusion criteria in NDR: *five blood glucose measurements within one year* and *two blood glucose measurements per year for five consecutive years*, might have questionable validity [30], and thus a sensitivity analysis was conducted excluding these (*n =* 3113). 

Possible multiplicative interactions between the healthy Nordic food index and BMI were investigated.

SAS^®^ statistical software (release 9.3, SAS Institute, Inc., Cary, NC, USA) was used for all statistical analyses. The PHREG procedure was used for the Cox proportional hazards models and the UNIVARIATE and FREQ procedures for the descriptive statistics. 

## 3. Results

During a median of 15 years of follow-up, 3269 women and 4097 men of the 55,060 cohort participants were diagnosed with T2D. Table 1 shows the baseline characteristics stratified by sex for all and according to the healthy Nordic food index score: lowest adherence (0–1 points), middle adherence (2–3 points), and highest adherence (4–6 points), respectively. A larger proportion of cases was present in the lowest adherence score category, compared to highest scores. Moreover, individuals with the highest scores also had the healthiest lifestyles for both men and women, *i.e.*, men and women with a high adherence score had more participation in sports and were less likely to be smokers compared to those with the poorest adherence to the index. 

**Table 1:** Baseline characteristics of all participants in the Danish Diet, Cancer and Health cohort in relation to adherence to the healthy Nordic food index (*n =* 55,060).

	Women (*n =* 28,953)								Men (*n =* 26,107)							
	All		0–1		2–3		4–6		All		0–1		2–3		4–6	
Cases, n / total participants, *n* (% cases)	3,269/28,953 (11%)		679/5,332 (13%)		1,570/13,477 (12 %)		1,020/10,144 (10 %)		4,097/26,107 (16%)		1,170/6,346 (18%)		1,888/11,570 (16%)		1,039/8,191 (13%)	
Characteristic	Median *or* %	P5-P95 *or* *n*	Median *or* %	P5-P95 *or* *n*	Median *or* %	P5-P95 *or* *n*	Median *or* %	P5-P95 *or* *n*	Median *or* %	P5-P95 *or* *n*	Median *or* %	P5-P95 *or* *n*	Median *or* %	P5-P95 *or* *n*	Median *or* %	P5-P95 *or* *n*
**Age (years)**	56	(50–64)	55	(50–64)	56	(50–64)	56	(50–64)	55	(50–64)	55	(50–64)	56	(50–64)	55	(50–64)
**School (%)**																
Short (≤7 years)	31%	(9025)	37%	(1,962)	34%	(4,250)	28%	(2,813)	35%	(9,040)	42%	(2,658)	35%	(4,030)	29%	(2,355)
Medium (8–10 years)	50%	(14,560)	49%	(2,607)	51%	(6,921)	50%	(5,033)	42%	(10,851)	42%	(2,646)	42%	(4,898)	40%	(3,308)
Long (≥11 years)	19%	(5368)	14%	(763)	17%	(2,307)	23%	(2,299)	24%	(6,216)	16%	(1,044)	23%	(2,646)	31%	(2,528)
**Smoking (%)**																
Never	44%	(12,617)	37%	(1,967)	42%	(5,718)	49%	(4,933)	26%	(6,740)	22%	(1,372)	25%	(2,911)	30%	(2,457)
Former	23%	(6,794)	19%	(999)	23%	(3,038)	27%	(2,757)	35%	(9,021)	28%	(1,803)	35%	(3,993)	39%	(3,227)
Current	33%	(9,542)	44%	(2,366)	35%	(4,722)	24%	(2,455)	40%	(10,346)	50%	(3,173)	40%	(4,670)	31%	(2,507)
**Alcohol intake**																
Abstainer (% = yes)	3%	(773)	4%	(195)	3%	(378)	2%	(200)	2%	(463)	2%	(148)	2%	(197)	1%	(118)
Alcohol * ≤12 g/day (women), ≤24 g/day (men)	58%	(16,856)	59%	(3,138)	57%	(7,736)	59%	(5,982)	56%	(14,567)	51%	(3,233)	56%	(6,436)	60%	(4,898)
Alcohol * >12 g/day (women), >24 g/day (men)	39%	(11,324)	37%	(1,999)	40%	(5363)	39%	(3,962)	42%	(11,077)	47%	(2,965)	43%	(4,937)	39%	(3175)
**BMI (kg/m^2^)**	24.8	(19.9–33.6)	24.8	(19.8–33.9)	24.8	(19.9–33.6)	24.6	(19.9–33.2)	26.1	(21.5–32.9)	26.5	(21.5–33.6)	26.2	(21.5–32.9)	25.8	(21.4–32.2)
**Waist circumference (cm)**	80	(67–103)	81	(67–104)	80	(67–103)	79	(67–102)	89	(81–113)	96	(82–115)	95	(82–113)	94	(81–111)
**Participate in sports (% = yes)**	59%	(16,960)	45%	(2,379)	57%	(7,637)	70%	(4,549)	49%	(12,766)	38%	(2379)	48%	(5567)	59%	(4821)
**Total energy intake including alcohol (mJ/day)**	8.5	(5.4–12.7)	7.1	(4.5–10.6)	8.2	(5.5–12.0)	9.6	(6.6–13.8)	10.7	(7.1–15.9)	9.4	(6.2–12.8)	10.6	(7.3–12.3)	11.9	(8.5–17.1)
**Intake of red meat (g/day)**	63	(27–120)	59	(27–111)	63	(27–118)	67	(26–128)	100	(46–190)	93	(44–175)	100	(47–188)	105	(47–200)
**Intake of processed meat (g/day)**	18	(4–50)	17	(4–48)	18	(4–51)	18	(3–41)	35	(9–89)	33	(10–87)	35	(9–90)	36	(9–89)

*Abbreviation*: P5: 5th percentile, P95: 95th percentile, d: day; *Among drinkers, Values are medians (for continuous variables) or percentages (for categorical variables). Values in brackets are 5–95 percentiles or numbers.

Adherence to the healthy Nordic food index was associated with a lower risk of T2D for both men and women (Table 2). In the model adjusted for potential confounding factors (model 2), a one-point higher in the index was associated with a 6% lower risk for women (HR, model 2: 0.94, 95%CI = 0.92–0.97) and a 9% lower risk for men (HR, model 2: 0.91; 95% CI: 0.89–0.93). When the index was assessed as a categorical variable, significant inverse associations were also found. Women with the highest adherence (5–6 points) had a 25% lower risk of T2D (HR, model 2: 0.75, 95%CI: 0.61–0.92) compared to women with the lowest adherence (0 points). For men, the association was even more pronounced with a 38% lower risk of T2D (HR, model 2: 0.62, 95%CI: 0.53–0.71) among men with the highest adherence (5–6 points). 

The confounder-adjusted estimates were less strong than the crude estimates. In the energy-adjusted model (model 3), a significant inverse association was also found, however, it was not significant when assessed categorical for women. Adjusting for BMI and WC (considered mediators) additionally moved the association towards unity, and the resulted in insignificant results for women.

When evaluating associations between the single index food items and T2D (Table 3), intakes of oatmeal and root vegetables above the cut-off values were associated with lower risk of T2D for both sexes. Moreover, for men significant associations were also found for intake of rye bread and cabbage.

In the sensitivity analysis where only “confirmed” cases (women = 1821, men = 2432) were included (Supplementary Table 1), the association between the index and T2D risk appeared slightly stronger. No significant interactions were found between BMI and the index when assessed in men and women (results not shown).

**Table 2:** Hazard ratio for the association between adherence to the healthy Nordic food index and T2D risk (*n =* 55,060)—The Danish Diet, Cancer and Health cohort.

	Women (*n =* 28,953)									Men (*n =* 26,107)								
	Cases (*n*)	Model 1 *		Model 2 **		Model 3 ***		Model 4 ****		Cases (*n*)	Model 1 *		Model 2 **		Model 3 ***		Model 4 ****	
		HR	95% CI	HR	95% CI	HR	95% CI	HR	95% CI		HR	95% CI	HR	95% CI	HR	95% CI	HR	95% CI
**Healthy Nordic food index** *(linear, per 1-unit increase)*	3269	0.92	0.90–0.94	0.94	0.92–0.97	0.96	0.94–0.99	0.97	0.94–1.00	4097	0.89	0.87–0.90	0.91	0.89–0.93	0.93	0.91–0.95	0.95	0.93–0.98
**Healthy Nordic food index** *(category)*																		
0	126	1.00	Reference	1.00	Reference	1.00	Reference	1.00	Reference	367	1.00	Reference	1.00	Reference	1.00	Reference	1.00	Reference
1	553	1.00	0.83–1.26	0.99	0.82–1.20	1.02	0.84-1.23	1.01	0.83–1.23	803	0.87	0.77–0.98	0.89	0.78–1.00	0.91	0.80–1.03	0.96	0.85–1.09
2	792	0.93	0.77–1.12	0.94	0.78–1.14	0.99	0.82–1.12	1.02	0.85–1.24	990	0.83	0.74–0.94	0.88	0.78–0.99	0.91	0.81–1.03	0.98	0.86–1.11
3	778	0.85	0.70–1.03	0.89	0.73–1.07	0.95	0.78–1.15	0.97	0.80–1.18	898	0.72	0.63–0.81	0.78	0.69–0.88	0.82	0.72–0.93	0.89	0.78–1.02
4	627	0.80	0.66–0.94	0.86	0.71–1.04	0.94	0.77–1.15	0.96	0.79–1.17	652	0.61	0.54–0.70	0.69	0.60–0.78	0.74	0.65–0.86	0.83	0.72–0.95
5–6	393	0.68	0.56–0.84	0.75	0.61–0.92	0.85	0.85–1.05	0.89	0.72–1.10	387	0.54	0.47–0.62	0.62	0.53–0.71	0.69	0.59–0.80	0.80	0.69–0.94
*P for trend* (linear)		*p* < 0.0001		*p* < 0.0001		*p* = 0.0100		*p* = 0.0436			*p* < 0.0001		*p* < 0.0001		*p* < 0.0001		*p* < 0.0001	

*Note:* All models are adjusted for age as underlying time scale and for "time under study"; *Abbreviations:* HR: Hazard Ratio. CI: Confidence intervals; * Crude; ** Adjusted for schooling level, participation in sports, smoking status, alcohol intake, red and processed meat; *** Additionally adjusted for total energy intake; **** Additionally adjusted for body mass index and waist circumference.

**Table 3:** Hazard ratio for the association between adherence to the single food items in the healthy Nordic food index and T2D risk (*n =* 55,060)—The Danish Diet, Cancer and Health cohort.

	Women (*n =* 28,953)								Men (*n =* 26,107)							
	Model 1 *		Model 2 **		Model 3 ***		Model 4 ****		Model 1 *		Model 2 **		Model 3 ***		Model 4 ****	
	HR	95% CI	HR	95% CI	HR	95% CI	HR	95% CI	HR	95% CI	HR	95% CI	HR	95% CI	HR	95% CI
**Healthy Nordic food items**																
**Fish**																
<Median	1.00	Reference	1.00	Reference	1.00	Reference	1.00	Reference	1.00	Reference	1.00	Reference	1.00	Reference	1.00	Reference
≥Median	1.00	0.93–1.08	1.00	0.93–1.08	1.03	0.96–1.11	0.98	0.91–1.06	1.02	0.96–1.08	1.02	0.96–1.09	1.04	0.97–1.11	1.02	0.95–1.08
**Cabbage**																
<Median	1.00	Reference	1.00	Reference	1.00	Reference	1.00	Reference	1.00	Reference	1.00	Reference	1.00	Reference	1.00	Reference
≥Median	0.92	0.86–0.99	0.98	0.91–1.06	0.99	0.92–1.07	0.99	0.92–1.06	0.84	0.78–0.90	0.89	0.83–0.95	0.89	0.83–0.96	0.92	0.86–0.99
**Rye bread**																
<Median	1.00	Reference	1.00	Reference	1.00	Reference	1.00	Reference	1.00	Reference	1.00	Reference	1.00	Reference	1.00	Reference
≥Median	0.94	0.87–1.02	0.91	0.84–0.99	0.94	0.86–1.02	0.96	0.88–1.04	0.84	0.79–0.89	0.79	0.74–0.85	0.82	0.76–0.87	0.89	0.84–0.96
**Oatmeal**																
<Median	1.00	Reference	1.00	Reference	1.00	Reference	1.00	Reference	1.00	Reference	1.00	Reference	1.00	Reference	1.00	Reference
≥Median	0.74	0.67–0.81	0.79	0.71–0.87	0.81	0.73–0.90	0.90	0.81–0.99	0.71	0.65–0.77	0.75	0.69–0.82	0.77	0.71–0.84	0.88	0.81–0.96
**Apples and pears**																
<Median	1.00	Reference	1.00	Reference	1.00	Reference	1.00	Reference	1.00	Reference	1.00	Reference	1.00	Reference	1.00	Reference
≥Median	1.02	0.95–1.09	1.01	0.94–1.09	1.04	0.96–1.12	1.03	0.96–1.11	0.98	0.92–1.04	1.02	0.96–1.09	1.04	0.97–1.11	0.97	0.91–1.04
**Root vegetables**																
<Median	1.00	Reference	1.00	Reference	1.00	Reference	1.00	Reference	1.00	Reference	1.00	Reference	1.00	Reference	1.00	Reference
≥Median	0.84	0.78–0.90	0.89	0.82–0.96	0.91	0.84–0.98	0.94	0.87–1.02	0.78	0.73–0.83	0.93	0.86–0.99	0.94	0.87–1.01	0.98	0.91–1.05

*Note:* All models are adjusted for age as underlying time scale and for "time under study"; *Abbreviations:* HR: Hazard Ratio. CI: Confidence intervals; * Crude; ** Adjusted for schooling level, participation in sports, smoking status, alcohol intake, red and processed meat; *** Additionally adjusted for total energy intake; **** Additionally adjusted for body mass index and waist circumference.

## 4. Discussion

In the present study, adherence with a healthy Nordic food index was associated with a lower risk of T2D. More specifically, women had a 6% lower risk and men had a 9% lower risk per one-point increment on the index score. A healthy Nordic food index score of 5−6 points (high adherence) was associated with a statistically significant 25% lower T2D risk for women and 38% for men compared to those with an index score of 0 points (poor adherence), when adjusted for potential confounders.

The strengths of the present study include the prospective design, large number of cases, detailed information about potential confounding factors, and long follow-up time. Further, a validated FFQ was used to assess food intake [23]. The study does, however, also have limitations: even though we used a validated FFQ as exposure measurement, measurement errors are likely, and further the diet of the participants was only assessed at baseline and might have changed during the long follow-up period. Furthermore, other healthy Nordic foods in the index could have been relevant to include, such as rapeseed oil. In studies of the Mediterranean diet, olive oil has shown to be associated with lower T2D risk [31]. It is possible that rapeseed oil would have similar beneficial effects [32]. However, it was not possible to include rapeseed oil due to the design of the FFQ. Finally, even though potential confounders were thoroughly considered, we cannot rule out the risk of residual confounding. 

The main hypothesis was that adherence to the healthy Nordic food index was associated with lower T2D risk, which was also the case in the present study. These findings might not be surprising given that each of the included food items are considered to carry health-enhancing effects [6,33,34]. Overall, none of the food items in the index seemed to be solely responsible for the association found, and thus this supports the idea that dietary patterns provides extra information compared to when studying individual foods [35]. The beneficial association found for *oatmeal, rye bread, root vegetables,* and *cabbages* might be explained by the high content of dietary fibre in these foods, as dietary fibers slow digestion and absorption, affecting the level of glucose as well as insulin sensitivity [36,37], which are markers related to development of T2D [5]. The lack of associations with *fish* and *apples/pears* in the present study is in congruence with the existing literature [38,39]. This may be that *fish* and *apples/pears* are not directly expected to have blood glucose-stabilizing effects, contrary to the other four food items in the index. 

Our results are also supported by findings from other studies, where a healthy Nordic diet has been investigated in relation to disease makers including markers of T2D risk [15,16,17,40,41]. For instance in the Swedish intervention study “NORDIET” where 33 mildly hypercholesterolemia participants were randomized to follow the NORDIET or a control diet [15]. The investigators found that adherence to NORDIET improved blood lipid profile (in respect to cholesterol) and reduced body weight in hypercholesterolemia participants, although there was no effect on blood pressure, plasma glucose, plasma triglyceride, or the inflammation marker C-reactive protein [15]. 

We expected that part of the association could be explained by high fibre content and low energy contents of the foods included in the index, and thus a lower risk of overweight and obesity, which are main risk factors of T2D [3,42]. Thus, we regarded BMI and WC as mediators rather than confounders in the association between the index and risk of T2D. As analyses including BMI and WC as covariates yielded less strong associations, it could be that the association is partly mediated by these. As most studies on Mediterranean diet consider BMI as potential confounder rather than mediator [43], we presented results both adjusted and unadjusted for BMI and WC in the tables to facilitate comparison. 

A small prospective study of less than 7000 participants with 541 incident T2D cases investigated the association between a healthy Nordic food index (The Baltic Sea Score) and risk of T2D. They found a tendency for an inverse association, but it was not statistically significant, probably due to the small study size [18]. To our knowledge, no previous large observational study has investigated a Nordic food in relation to incidence of T2D particularly. However, the regional Mediterranean diet has been related to lower risk of T2D in several observational studies [8,10,11]. Thus both a healthy Nordic diet and the Mediterranean diet seems to be related to lower risk of T2D. Previous studies have indicated that it might be difficult for people to comply with a “foreign” diet [13]. Therefore, it could be advocated that a more regional diet should be promoted. Thus, this could not only enhance compliance, but also help in conserving the environment and cultural diversity [6].

The result of the present study may not be entirely generalizable. The cohort included only people aged 50-64 and thus may represent a population with a higher T2D risk than the entire population. It is therefore likely that the associations found are stronger than expected for the entire population. 

## 5. Conclusions

In conclusion, we found an inverse association between a healthy Nordic index and risk of T2D for both men and women. Thus, healthy aspects of the Nordic diet may play a role in the prevention of T2D. Promoting regional diets might be an effective and sustainable way of improving public health.

## Supplementary Materials

Supplementary materials can be accessed at: http://www.mdpi.com/2072-6643/7/10/5418/s1.
