# Supplementary material for: Adherence to a Healthy Nordic Food Index Is Associated with a Lower Risk of Type-2 Diabetes—The Danish Diet, Cancer and Health Cohort Study
Source: Nutrients. 2015 Oct 21;7(10):8633–44. doi: 10.3390/nu7105418 (PMC4632438; doi:10.3390/nu7105418)
Supplement: Supplementary file 1 [file nutrients-07-05418-s001.docx]

**Supplementary Material**

**Table S1.** Hazard ratio for the association between the healthy Nordic food index and risk of T2D, excluding cases diagnosed from blood glucose measurements
(*n =* 51,947).

|  | **All Women (*n =* 28,953)** | | | **Excluding Those Diagnosed Based on Blood Glucose Measurements ¥**  **Women (*n =* 27,505)** | | | **All Men (*n =* 26,107)** | | | **Excluding Those Diagnosed Based on Blood Glucose Measurements ¥**  **Men (*n =* 24,442)** | | |
| --- | --- | --- | --- | --- | --- | --- | --- | --- | --- | --- | --- | --- |
|  | **Model 3 ***** | | | **Model 3 ÷ 2 g/5 year & 5 g/1 year** | | | **Model 3 ***** | | | **Model 3 ÷ 2 g/5 year & 5 g/1 year** | | |
|  | **Cases (*n*)** | **HR** | **95% CI** | **Cases (*n*)** | **HR** | **95% CI** | **Cases (*n*)** | **HR** | **95% CI** | **Cases (*n*)** | **HR** | **95% CI** |
| **Healthy Nordic food index** *(linear, per 1-unit increase)* | 3269 | 0.96 | 0.94–0.99 | 1821 | 0.95 | 0.92–0.99 | 4097 | 0.93 | 0.91–0.95 | 2432 | 0.91 | 0.89–0.94 |
| **Healthy Nordic food index** *(category)* |  |  |  |  |  |  |  |  |  |  |  |  |
| 0 | 126 | 1.00 | Reference | 72 | 1.00 | Reference | 367 | 1.00 | Reference | 236 | 1.00 | Reference |
| 1 | 553 | 1.02 | 0.84–1.23 | 317 | 1.02 | 0.79–1.32 | 803 | 0.91 | 0.80–1.03 | 478 | 0.85 | 0.73–0.99 |
| 2 | 792 | 0.99 | 0.82–1.12 | 446 | 0.99 | 0.77–1.27 | 990 | 0.91 | 0.81–1.03 | 623 | 0.91 | 0.78–1.06 |
| 3 | 778 | 0.95 | 0.78–1.15 | 443 | 0.96 | 0.75–1.24 | 898 | 0.82 | 0.72–0.93 | 511 | 0.76 | 0.64–0.89 |
| 4 | 627 | 0.94 | 0.77–1.15 | 344 | 0.93 | 0.72–1.22 | 652 | 0.74 | 0.65–0.86 | 377 | 0.70 | 0.59–0.84 |
| 5–6 | 393 | 0.85 | 0.85–1.05 | 89 | 0.78 | 0.59–1.04 | 387 | 0.69 | 0.59–0.80 | 207 | 0.60 | 0.49–0.74 |
| *P for trend* ((linear) |  | *p* = 0.0100 | |  | *p* = 0.0090 | |  | *p* < 0.0001 | |  | *p* < 0.0001 | |

*Note:* All estimates are adjusted for age as underlying time scale and for “time under study” as well as schooling level, participation in sports, smoking status, alcohol intake, red and processed meat, and total energy intake; *** Adjusted for schooling level, participation in sports, smoking status, alcohol intake, red and processed meat, and total energy intake; *Abbreviations:* HR: Hazard Ratio. CI: Confidence intervals; ¥ Excluded those included as cases because they had either two blood glucose measurements per year for five consecutive years or five blood glucose measurements within one year.

© 2015 by the authors; licensee MDPI, Basel, Switzerland. This article is an open access article distributed under the terms and conditions of the Creative Commons by Attribution (CC-BY) license (http://creativecommons.org/licenses/by/4.0/).
